# BMP9 maintains the phenotype of HTR-8/Svneo trophoblast cells by activating the SDF1/CXCR4 pathway

**DOI:** 10.1186/s12860-023-00487-0

**Published:** 2023-08-07

**Authors:** Xue Yang, Lingling Ren, Xiang Chen, Ying Pang, Baoxia Jia, Jing Sun, Xiaofang Quan

**Affiliations:** Obstetrics department of Weapon Industry 521 Hospital, NO.12, East Zhangba Road, Xi’an, Shannxi 710065 China

**Keywords:** BMP9, SDF1/CXCR4 axis, HTR-8/SVneo cells, Preeclampsia

## Abstract

**Background:**

Bone morphogenetic protein 9 (BMP9) has been shown to regulate processes such as angiogenesis, endothelial dysfunction, and tumorigenesis. However, the role of BMP9 in preeclampsia (PE) is unclear. The purpose of this study was to investigate the role and mechanism of BMP9 in PE.

**Methods:**

The effects of BMP9 on the viability, migration and invasion of HTR-8/Svneo cells were investigated by CCK-8 assay, wound healing assay and Transwell invasion assay. The effect of BMP9 on apoptosis of HTR-8/Svneo cells was detected by flow cytometry. Plasma levels of BMP9, SDF1 and CXCR4 were detected by ELISA kit. qRT-PCR and Western blot were used to detect the expression levels of each gene in the cells.

**Results:**

Overexpression of BMP9 promoted the proliferation and migration of trophoblast cells and inhibited apoptosis. Knockdown of BMP9 had the opposite effect. The levels of BMP9, SDF1 and CXCR4 in the plasma of PE patients were down-regulated, and BMP9 was positively correlated with the levels of SDF1 and CXCR4. BMP9 also significantly upregulated the mRNA and protein levels of SDF1 and CXCR4 in HTR-8/SVneo cells. Further mechanistic studies found that BMP9 promoted the migration and invasion of HTR-8/SVneo cells and inhibited apoptosis by activating the SDF1/CXCR4 pathway.

**Conclusion:**

We demonstrate for the first time that BMP9 promoted the migration and invasion of HTR-8/SVneo cells and inhibits apoptosis by activating the SDF1/CXCR4 pathway. This suggests that BMP9 may be a biomarker molecule for PE.

**Supplementary Information:**

The online version contains supplementary material available at 10.1186/s12860-023-00487-0.

## Background

Preeclampsia (PE) is a pregnancy-specific disorder that usually occurs after 20 weeks of gestation and is characterized by hypertension and endothelial dysfunction [1]. The pathogenesis of PE involves two stages, including impaired spiral artery remodeling due to insufficient placental trophoblast invasion and endothelial dysfunction due to the release of anti-angiogenic factors in the ischemic placenta [2]. Its morbidity involves multiple end-organ damage and increases the risk of maternal and neonatal death [3]. In addition, a history of PE was considered a risk factor for future cardiovascular events. Women with a history of PE have a significantly increased risk of cardiovascular disease later in life [4]. Therefore, seeking new targets is crucial for the diagnosis and treatment of PE. This study was based on human trophoblast cells HTR-8/Svneo to explore the potential role of BMP9 in PE.

Bone morphogenetic protein 9 (*BMP9*), a member of the TGF-β family, has shown unique roles in bone formation, angiogenesis, glucose metabolism and tumorigenesis [5]. BMP9 is a vascular quiescence and endothelial protective factor, which is important in cardiovascular disease [6]. Studies have shown that circulating BMP9 is associated with the prevalence of cardiovascular disease, and the level of circulating BMP9 in patients with essential hypertension is lower than in healthy individuals [7]. Decreased levels of BMP9 are independently and significantly associated with essential hypertension, and BMP9 is considered a serum biomarker for essential hypertension [8]. In pulmonary hypertension, BMP9 acts directly on the endothelium to reverse the pathological state of pulmonary hypertension by preventing apoptosis and enhancing vascular stability [9]. Administration of recombinant human bone morphogenetic protein 9 (rhBMP9) can effectively attenuate bleomycin-induced pulmonary hypertension [10]. It is well known that hypertension is one of the distinguishing features of PE. The antihypertensive effect of BMP9 may be equally effective in gestational hypertension and benefit the development of PE. In the existing studies, the role and mechanism of BMP9 in PE is still unclear.

Stromal cell-derived factor 1 (*SDF1*) is a member of the chemokine subfamily, also known as *CXCL12* [11]. SDF1 plays a key role in a variety of diseases, such as transporting hematopoietic stem cells, guiding cell migration, and promoting angiogenesis [12]. SDF1 has been identified to induce neovascularization in the motor system. Overexpression of SDF1 significantly enhanced the proliferation and migration of primary nucleus pulposus cells, as well as the invasion and angiogenesis of vascular endothelial cells, and accelerated angiogenesis [13]. Furthermore, SDF1 can enhance crosstalk between trophoblast and decidual cells, regulating trophoblast function and uterine spiral artery remodeling [14]. This has important implications for a variety of pregnancy-related diseases. The investigation found that the serum level of SDF1 was differentially expressed between PE patients and healthy subjects, thus it was considered as a potential marker for PE diagnosis [15]. CXCR4 is a G protein-coupled receptor and acts as a receptor for SDF1 [11]. CXCR4 also showed an important role in PE. The mRNA and protein expression levels of CXCR4 in trophoblast cells from patients with severe preeclampsia were lower than those in normal patients, and CXCR4 was associated with trophoblast apoptosis [16]. SDF1 and its receptor CXCR4 together regulate the function of trophoblast cells, decidual stromal cells, and endometrial epithelial cells, delaying the progression of pregnancy complications [17, 18]. Therefore, it is instructive to explore the role of SDF1/CXCR4 in PE.

In the present study, we examined the serum levels of BMP9, SDF1 and CXCR4 in the serum of PE patients with healthy subjects as controls. Next, we explored the effect of BMP9 on the proliferation, migration, invasion and apoptosis of HTR-8/Svneo trophoblast cells. In addition, this study also explored the mechanism by which BMP9 regulates HTR-8/Svneo cells. The purpose of this study is to provide theoretical basis and new directions for the diagnosis and treatment of PE.

## Materials and methods

### Cell culture

Human trophoblast cells HTR-8/SVneo were purchased from the American Type Culture Collection (ATCC, USA). Cells were cultured in RPMI 1640 medium (Procell, Wuhan, China) containing 10% fetal bovine serum (FBS; Gibco, MD), 1% penicillin and streptomycin. Cells were incubated at 37 °C and 5% CO2 and routinely passaged every 3 days.

### Cell transfection

pcDNA3.1-NC (empty vector), pcDNA3.1-BMP9, si-NC (empty vector), si-BMP9 and si-SDF1 were purchased from GeneChem (Shanghai, China). Cells were transfected with plasmids using Lipofectamine 2000 (Invitrogen, Carlsbad, USA). After 24 h of transfection, the cells were tested for transfection efficiency and subsequent experiments were performed.

### Collection of tissues

This study selected pregnant women who underwent antenatal care and delivery in the obstetrics department of Weapon Industry 521 Hospital from January 2020 to December 2021, including 20 PE patients and 20 healthy pregnant women. All patients were primigravida with a gestational age of 34–39 weeks. In addition to PE, patients with PE had no other medical and surgical complications. Healthy pregnant women selected as controls had gestational age and body mass index similar to PE patients. Pregnant blood was collected for centrifugation, and plasma was retained for subsequent studies. All subjects signed informed consent. The study was approved by the Weapon Industry 521 Hospital Ethics Committee.

### Enzyme-linked immunosorbent assay (ELISA)

Plasma levels of BMP9, SDF1 and CXCR4 were determined using ELISA kits. The kit details are as follows: Human BMP9 ELISA Kit (ab267648, abcam, UK), Human CXCL12/SDF-1 ELISA Kit (PC205, Beyotime, China), Human CXCR4 ELISA Kit (TW14529, Tongwei Industry, China). All operating steps in the experiment were carried out in accordance with the reagent manufacturer’s instructions.

### CCK-8 assay

In this experiment, Cell Counting Kit-8 (C0038, Beyotime, China) was used to detect the viability of HTR-8/Svneo cells. HTR-8/Svneo (1 × 10^4^) cells were seeded in 96-well with culture plates, and CCK-8 assays were performed at 0, 12 h, 24 h, 48 h after seeding. Add 20 µl of CCK-8 solution to each well and incubate for 2 h. Use a microplate reader to measure the absorbance of each well at 450 nm.

### Wound healing test

Select cells (1 × 10^6^) at 90% growth density and use a 200 µL micropipette to scrape the cells to make an even scratch. The damaged cells were washed with PBS and cultured in the incubator for 24 h. Cells were observed for wound healing at 0 and 24 h using a microscope.

### Transwell invasion assay

First, Transwell chambers (Corning, USA) were coated with Matrigel (BD Biosciences, Germany). Approximately 1 × 10^5^ transfected HTR- 8/Svneo cells in 200 µl serum- free medium were seeded into the top compartment of transwell chambers with- out or with Matrigel. After 24 h of incubation, cells in the lower chamber were fixed with 4% paraformaldehyde and stained with crystal violet (C0121, Beyotime, China) for 15 min. Cell invasion was observed and recorded under a microscope.

### Apoptosis

In this experiment, Annexin V-FITC/PI apoptosis detection kit (40302ES20, Qcbio, China) was used to determine the apoptosis level of cells. After cells were trypsinized without EDTA, they were centrifuged and collected. After the cell pellet was washed twice with PBS, the cells were resuspended in 100 µL of 1×Binding Buffer. Add 5 µl Annexin V-FITC and 10 µl PI to each 5 × 105 cells, and react at room temperature for 15 min. Add 400 µL of 1×Binding Buffer to the cells again to prepare the test sample. The rate of apoptosis was assessed using a FACS flow cytometry system (BD FACS Canto II USA).

### qRT-PCR

Total RNA was extracted from HTR-8/SVneo cells using TRIzol reagent (Invitrogen). Next, RNA was reverse transcribed into cDNA using a reverse transcription kit (RR047A, Takara Bio Inc, Otsu). Quantitative PCR was performed using the DNA Engine Opticon™ Real-Time PCR System (MJ Research, MA). Gene expression levels were quantified using the 2^−ΔΔCT^ method. The primer sequences were as follows: BMP9: Forward: 5’- CCTGGGCACAACAAGGAC − 3’; Reverse: 5’- CCTTCCCTGGCAGTTGAG − 3’.

SDF1: Forward: 5′-FCTCCGCTGTCACCTTCCC-3′;

Reverse: 5’-RTGTGCCCTTCAGATTGTAGCC-3’.

CXCR4: Forward: 5’- CCG AGG CCC TAG CTT TCT TC -3’;

Reverse: 5’-GAG GAT CTT GAG GCT GGA CC-3’.

GAPDH: Forward: 5’- CGCTCTCTGCTCCTCCTGTTC − 3’,

Reverse: 5’-ATCCGTTGACTCCGACCTTCAC-3’.

### Western blot

In this experiment, lysis buffer (P0013J, Beyotime, China) was used to extract the protein in the cells, and the concentration of the extracted protein was quantified. Protein samples were separated using 10% SDS-polyacrylamide gel electrophoresis and transferred to polyvinylidene fluoride (PVDF) membranes. Membranes were blocked with 5% nonfat milk. Membranes were incubated overnight at 4 °C with diluted primary antibodies. Then, the membrane was incubated with the diluted secondary antibody for 2 h at room temperature. Protein expression levels were detected using Enlight TM-PLUS kit (17,100, Engreen Biosystem, China).

### Statistical analysis

Data are presented as the mean of three independent biological replicates. Data were processed by GraphPad software, and differences between 2 groups were explored using unpaired *t*-test, and differences between more than two groups were analyzed by one-way ANOVA combined with Tukey’s test. *P* < 0.05 was considered statistically significant.

## Results

### BMP9 promoted HTR-8/SVneo cell proliferation, migration and invasion

**Fig. 1:**
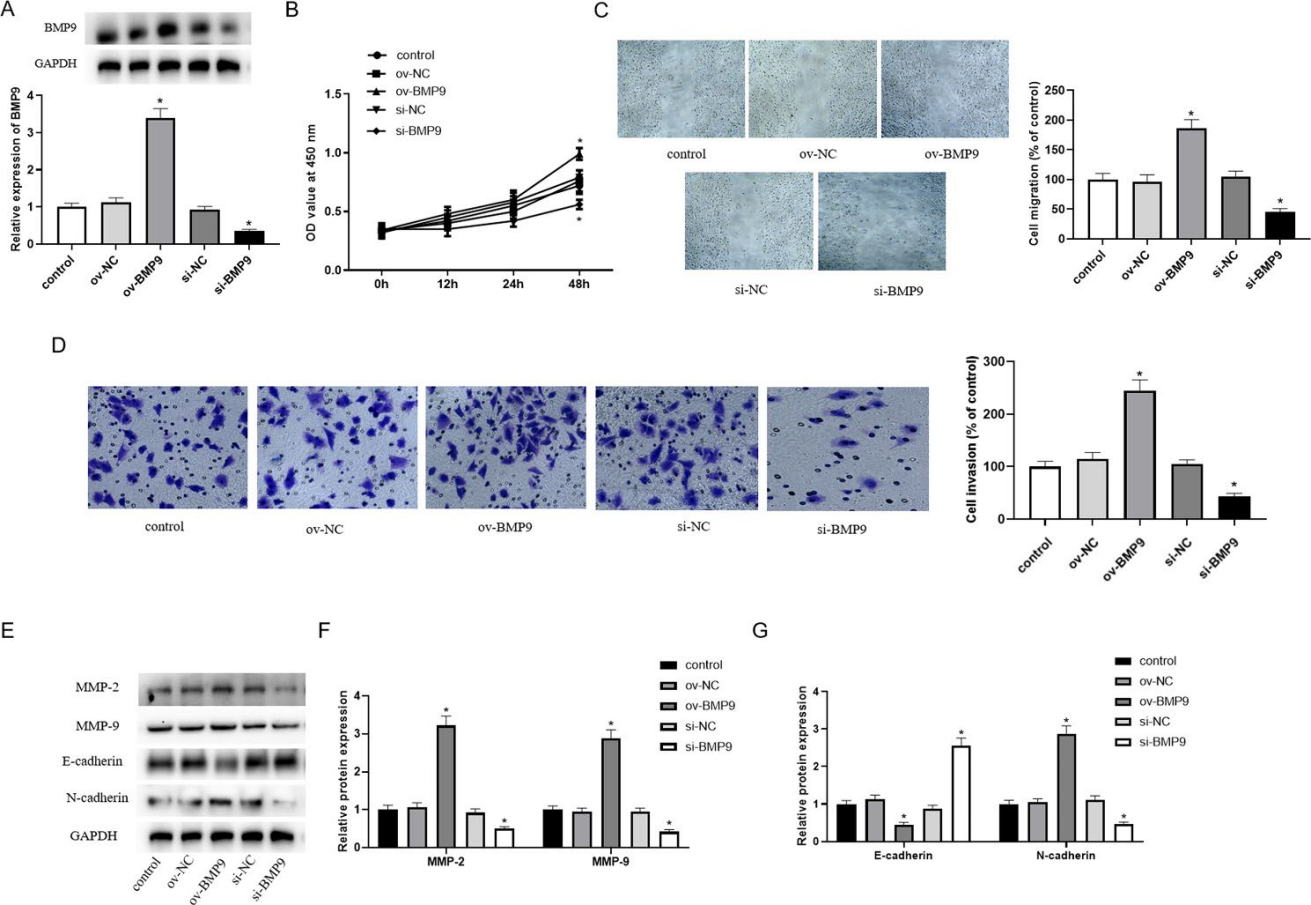
BMP9 promoted proliferation, migration and invasion of HTR-8/SVneo cells. After transfection of pcDNA3.1-BMP9 and si-BMP9 plasmids for 24 h, the following indexes were measured. (**A**) The expression of of BMP9 in HTR-8/SVneo cells. (**B**) The proliferation of HTR- 8/SVneo cells was evaluated by CCK8 assay. (**C**) The migratory capacity of cells was determined by wound healing assay. (**D**) The invasive ability of cells was detected by transwell assay. (**E**) Western blot was used to detect the expression levels of migration and invasion-related proteins (MMP-2, MMP-9, E-cadherin and N-cadherin). (**F**) The expression of MMP-2 and MMP-9 in HTR-8/SVneo cells. (**G**) The expression of E-cadherin and N-cadherin in HTR-8/SVneo cells. ^*^ means *P < 0.05* compared to ov-NC, ^#^ means *P < 0.05* compared to si-NC.

First, we examined the role of BMP9 in HTR-8/SVneo cells. The transfection results showed that BMP9 was successfully overexpressed or knocked down in HTR-8/SVneo cells (Fig. 1A). Overexpression of BMP9 resulted in significantly enhanced proliferation of HTR-8/SVneo cells, whereas knockdown of BMP9 resulted in decreased proliferation of HTR-8/SVneo cells (Fig. 1B). Overexpression of BMP9 significantly increased cell migration and invasion ability, while inhibition of BMP9 decreased cell migration distance and invasion rate (Fig. 1C and D). In addition, BMP9 significantly up-regulated the protein levels of MMP-2, MMP-9, and N-cadherin, and down-regulated the protein levels of E-cadherin. However, the effect of knockdown of BMP9 on HTR-8/SVneo cells showed the opposite trend (Fig. 1E and G).

### BMP9 inhibited apoptosis of HTR-8/SVneo cells

**Fig. 2:**
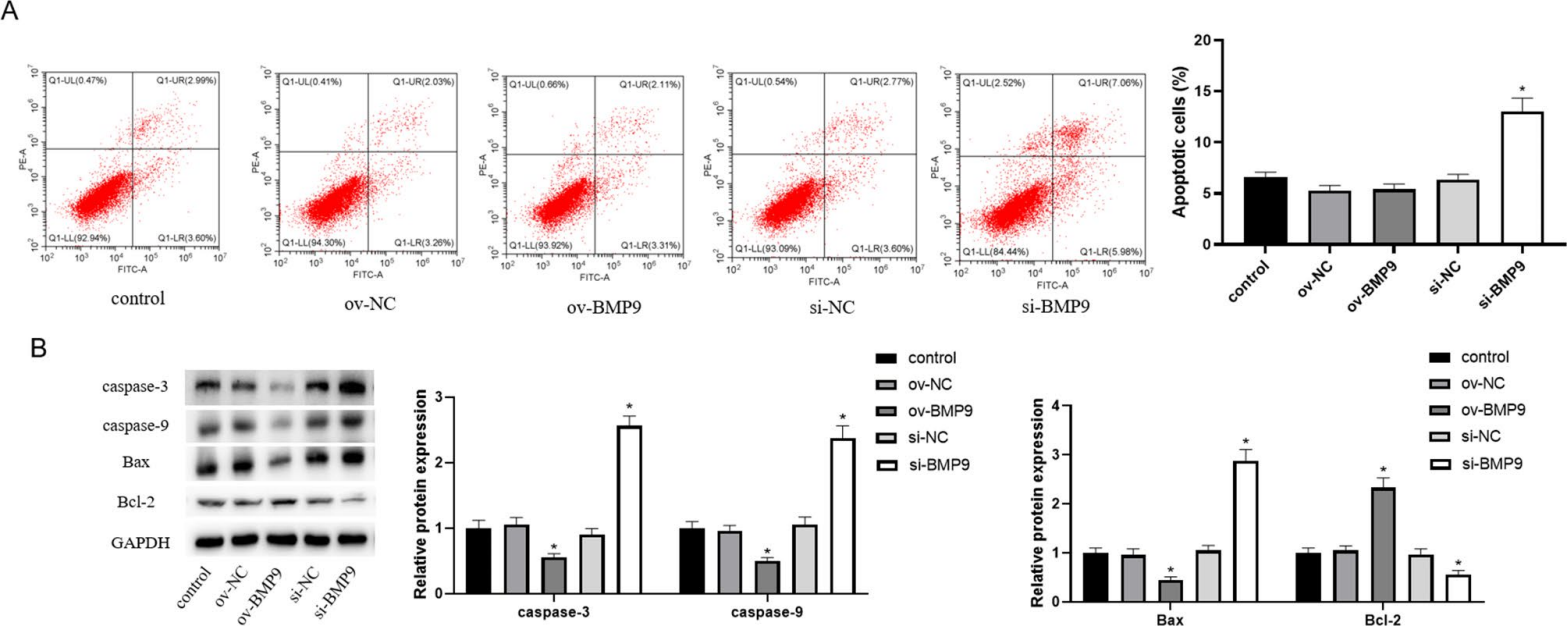
BMP9 inhibited apoptosis of HTR-8/SVneo cells. After transfection of pcDNA3.1-BMP9 and si-BMP9 plasmids for 24 h, the level of apoptosis was measured. (**A**) Apoptosis ratio was detected by flow cytometry. (**B**) The levels of apoptosis-related proteins (caspase-3, caspase-9, Bax and Bcl-2) were detected by western blot. ^*^ means *P < 0.05* compared to ov-NC, ^#^ means *P < 0.05* compared to si-NC.

Next, we examined the effect of BMP9 on apoptosis in HTR-8/SVneo cells. Flow cytometry results showed that overexpression of BMP9 significantly decreased the apoptosis ratio of cells, and knockdown of BMP9 increased HTR-8/SVneo cell apoptosis (Fig. 2A). In addition, the detection results of apoptosis-related proteins showed that overexpression of BMP9 down-regulated the expression of caspase-3, caspase-9 and Bax, and increased the protein expression level of Bcl-2 (Fig. 2B). Knockdown of BMP9 had the opposite effect.

### BMP9 positively regulated the expression of SDF1 and CXCR4

**Fig. 3:**
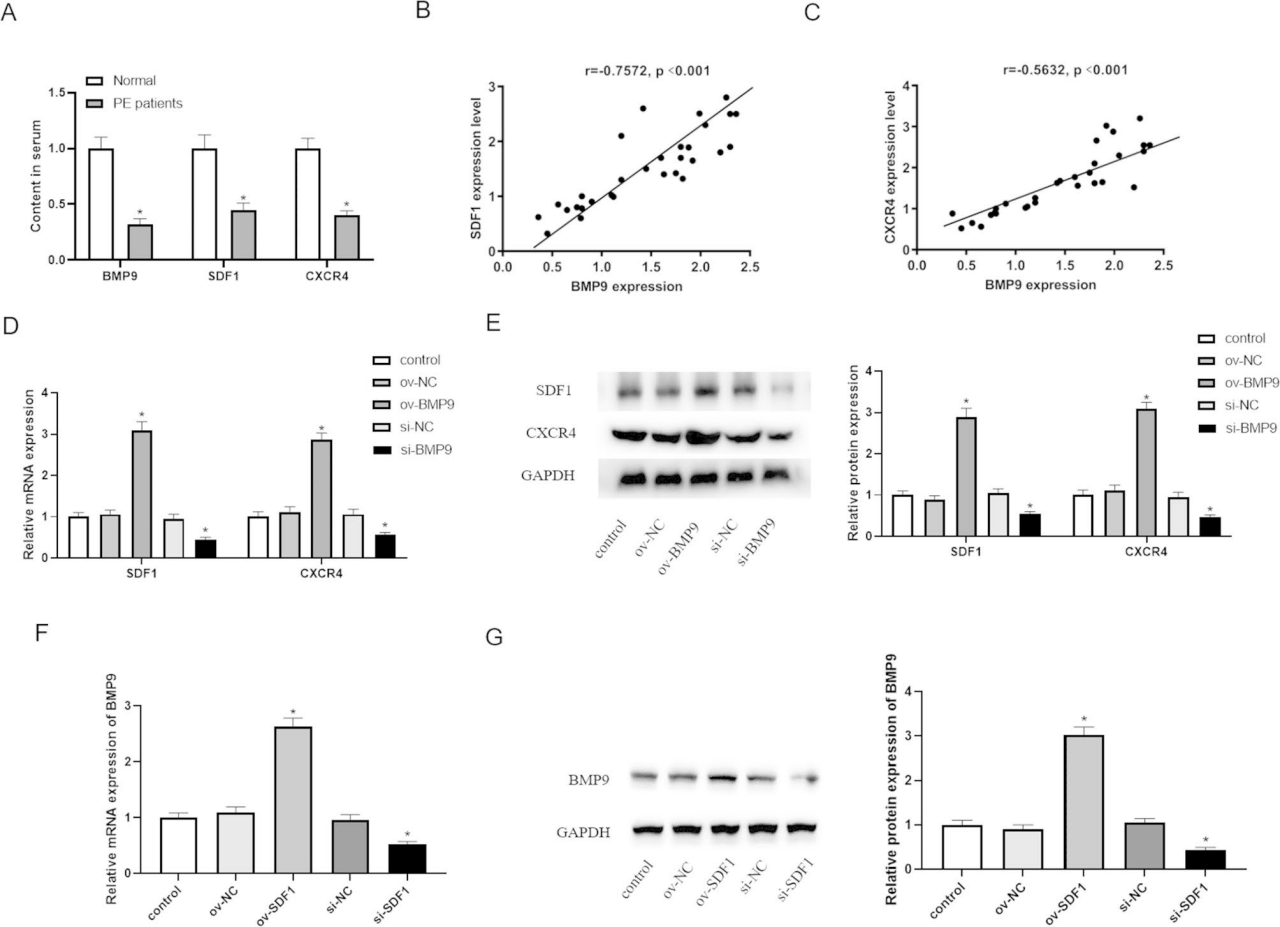
BMP9 positively regulates the expression of SDF1 and CXCR4. (**A**) Serum levels of BMP9, SDF1 and CXCR4 in healthy subjects and PE patients. ^*^ means *P < 0.05* compared to Normal. (**B**) Correlation analysis of BMP9 and SDF1. (**C**) Correlation analysis of BMP9 and CXCR4. (**D**) mRNA levels of SDF1 and CXCR4 when BMP9 was overexpressed and knocked down. (**E**) Protein levels of SDF1 and CXCR4 when BMP9 was overexpressed and knocked down. (**F**) mRNA levels of BMP9 when SDF1 was overexpressed and knocked down. (**G**) Protein levels of BMP9 when SDF1 was overexpressed and knocked down.^*^ means *P < 0.05* compared to ov-NC, ^#^ means *P < 0.05* compared to si-NC.

To verify the potential mechanism of BMP9 in PE, we initially detected the levels of BMP9, SDF1 and CXCR4 in the serum of clinical patients. Serum levels of BMP9, SDF1 and CXCR4 were significantly reduced in PE patients compared with healthy subjects (Fig. 3A). The results of correlation analysis showed that BMP9 was positively correlated with the levels of *SDF1* and *CXCR4* (Fig. 3B C). Cell experiments showed that overexpression of BMP9 up-regulated the mRNA and protein levels of *SDF1* and *CXCR4* in HTR-8/SVneo cells, and inhibition of BMP9 down-regulated the mRNA and protein levels of *SDF1* and *CXCR4* (Fig. 3D and E). As expected, overexpression of SDF1 upregulated the mRNA and protein levels of BMP9 in HTR-8/SVneo cells, while knocking down SDF1 downregulated the mRNA and protein expression of BMP9 (Fig. 3F and G). The above results showed that BMP9 positively regulates the expression of SDF1 and CXCR4.

### BMP9 promoted HTR-8/SVneo cell proliferation, migration and invasion by activating the SDF1/CXCR4 pathway

**Fig. 4:**
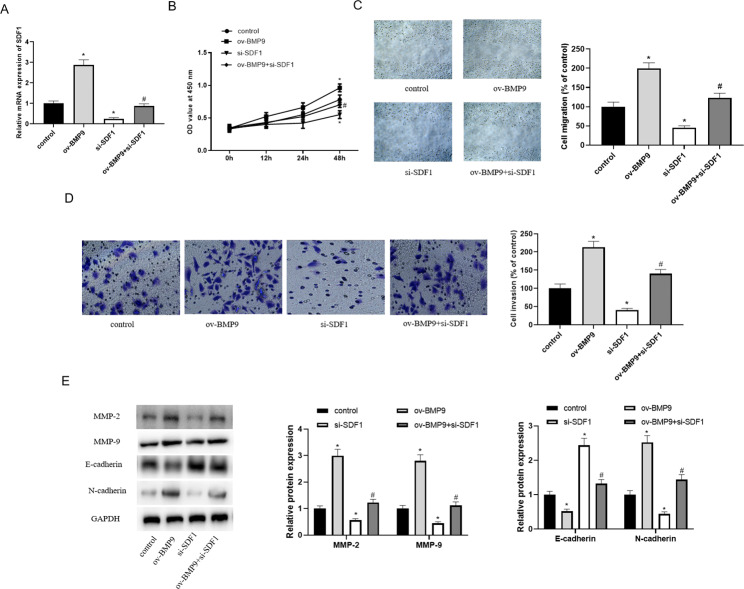
BMP9 regulates the proliferation, migration and invasion of HTR-8/SVneo cells by regulating the SDF1/CXCR4 pathway. After transfection of pcDNA3.1-BMP9 and si-SDF1 plasmids for 24 h, the following indexes were measured. (**A**) RT-PCR was used to detect the transfection efficiency of cells. (**B**) The proliferation of HTR- 8/SVneo cells was evaluated by CCK8 assay. (**C**) Wound healing assay was used to measure cell migration ability. (**D**) The invasive ability of cells was detected by transwell assay. (**E**) Western blot was used to detect the expression levels of migration and invasion-related proteins (MMP-2, MMP-9, E-cadherin and N-cadherin). ^*^ means *P < 0.05* compared to control, ^#^ means *P < 0.05* compared to ov-BMP9.

Next, we verified the mechanism by which BMP9 regulates the proliferation, migration and invasion of HTR-8/SVneo cells by overexpressing BMP9 and knocking down *SDF1*. The transfection results showed good efficiency of cell overexpression and knockdown (Fig. 4A). Overexpression of BMP9 significantly increased the proliferation of HTR-8/SVneo cells, and knockdown of SDF1 reversed the promotion of BMP9 overexpression on HTR8/SVneo cell proliferation (Fig. 4B). In addition, BMP9 enhanced cell migration and invasion ability, and knockdown of SDF1 reversed the promoting effect of overexpression of BMP9 on cell phenotype (Fig. 4C and D). Overexpression of BMP9 also up-regulated the protein levels of MMP-2, MMP-9 and N-cadherin, and down-regulated the protein level of E-cadherin. While knockdown of SDF1 decreased the expression of CXCR4, MMP-2, MMP-9 and N-cadherin, as well as increased the expression of E-cadherin (Fig. 4E). Inhibition of SDF1 reversed the effects of overexpression of BMP9. The above results showed that BMP9 promotes the proliferation, migration and invasion of HTR-8/SVneo cells by activating the SDF1/CXCR4 pathway.

### BMP9 inhibited apoptosis of HTR-8/SVneo cells by activating the SDF1/CXCR4 pathway

**Fig. 5:**
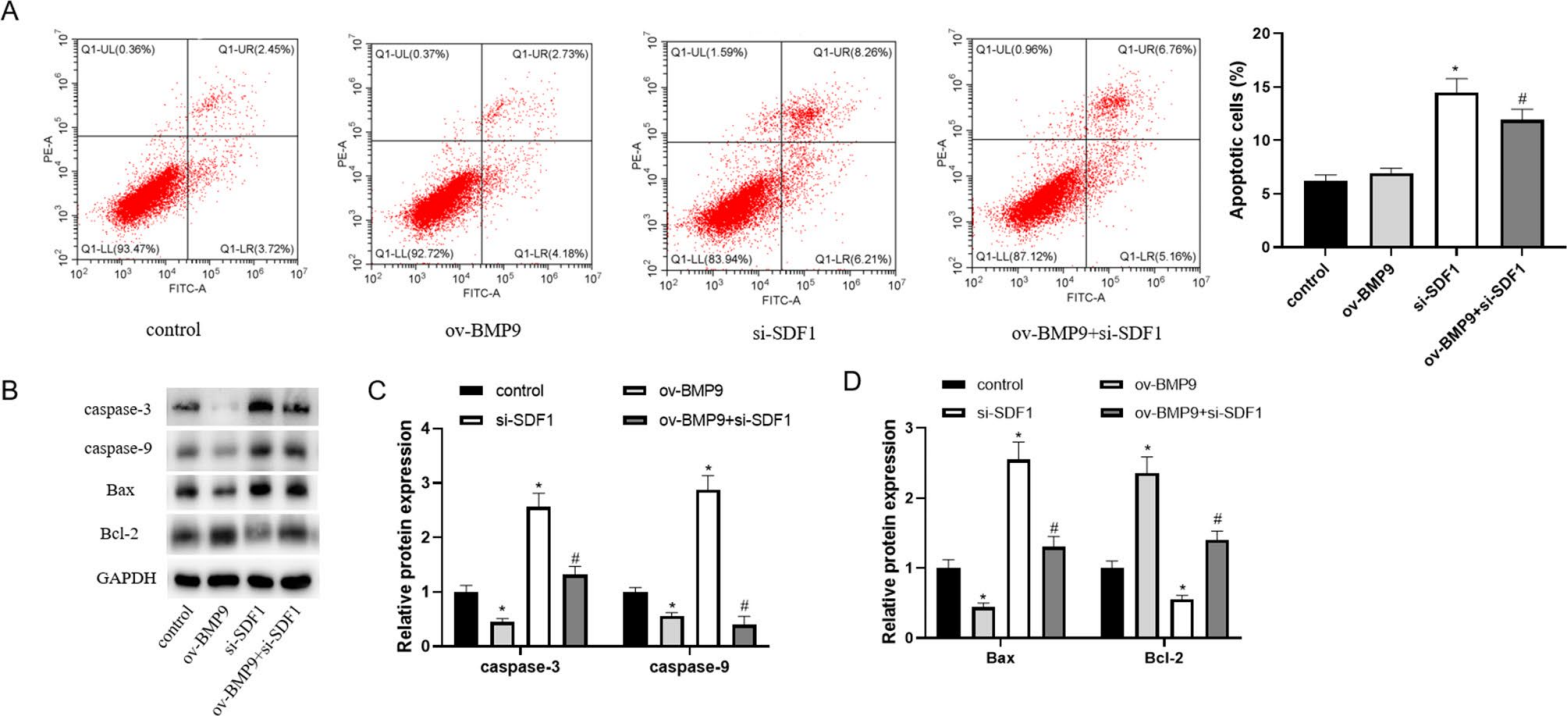
BMP9 regulates HTR-8/SVneo cell apoptosis by regulating the SDF1/CXCR4 pathway. After transfection of pcDNA3.1-BMP9 and si-SDF1 plasmids for 24 h, the level of apoptosis was measured. (**A**) Apoptosis ratio was tested by flow cytometry. (**B-D**) The levels of apoptosis-related proteins (caspase-3, caspase-9, Bax and Bcl-2) were detected by western blot. ^*^ means *P < 0.05* compared to control, ^#^ means *P < 0.05* compared to ov-BMP9.

Flow cytometry results showed that overexpression of BMP9 significantly reduced the apoptotic ratio of HTR-8/SVneo cells, and knockdown of SDF1 reversed the decrease in apoptotic ratio caused by overexpression of BMP9 (Fig. 5A). The detection results of apoptosis-related proteins showed that overexpression of BMP9 down-regulated the expression of caspase-3, caspase-9 and Bax, and increased the protein expression level of Bcl-2. Knockdown of SDF1 reversed the effects of overexpression of BMP9. The protein levels of caspase-3, caspase-9 and Bax were up-regulated in the ov-BMP9 + si-SDF1 group relative to the ov-BMP9 group (Fig. 5B and D).

## Discusssion

During placentation, trophoblast cells invade the decidua and myometrium, further remodeling the uterine spiral arteries. When trophoblast invasion is insufficient, placental function is impaired, triggering various obstetric syndromes including PE [19]. Although extravillous trophoblasts are regulated by different parental cell types during invasion, trophoblasts are still the predominant cell type regulating PE. Because these cells are more or less interacting with extravillous trophoblasts [20]. The occurrence of PE is attributed to insufficient invasion of trophoblast cells. Therefore, exploring the metastatic and invasive properties of trophoblast cells is crucial for the progression of PE. Studies have shown that promoting the migration and invasion of rat placental trophoblast cells helps control gestational hypertension [21]. Invasive deficiency of trophoblast cells and impaired spiral arterial remodeling underlie fetal growth restriction and preeclampsia-like symptoms in hypertensive rats [22]. In addition, trophoblast apoptosis also regulates the progression of PE. In preeclampsia and intrauterine growth restriction pregnancies, trophoblast apoptosis is elevated [23]. In the present study, we identified a possible beneficial role of BMP9 in PE by investigating the migratory, invasive and apoptotic properties of HTR-8/Svneo trophoblasts.

An important function of BMP9 is to regulate angiogenesis. In vascular development, BMP9 induces the expansion and sprouting of human embryonic stem cell-derived vascular cells, which in turn promotes angiogenesis [24]. The specificity of BMP9 in angiogenesis confers a unique role in regulating hypertension and endothelial dysfunction. BMP9 can inhibit pulmonary hypertension by directly or indirectly affecting the function of endothelial cells [9]. In addition, existing reports have also shown that BMP9 has a role in regulating tumor progression. In bladder cancer, upregulation of BMP9 promotes the proliferation and migration of bladder cancer cells [25]. BMP9 also induces epithelial-to-mesenchymal transition in hepatoma cells [26]. During pregnancy, the migratory and invasive abilities of trophoblast cells are important for the normal development of the placenta. Given that the biological behavior of trophoblast cells is similar to that of cancer cells, we speculate that BMP9 may play a role in suppressing the trophoblast phenotype. In this study, we confirmed that BMP9 levels were downregulated in the serum of PE patients. Overexpression of BMP9 significantly promoted the proliferation, migration and invasion of HTR-8/SVneo cells, as well as inhibited cell apoptosis. Silencing of BMP9 inhibited the proliferation, migration and invasion of HTR-8/SVneo cells, and promoted cell apoptosis. BMP9 may be an important regulator of PE.

MMP2 and MMP9 are important factors leading to the invasion of trophoblasts into the maternal vasculature and are involved in the remodeling of the placenta and uterine arteries [27]. Decreased MMP-2 and MMP-9 can decrease angiogenesis, reduce trophoblast invasion of spiral arteries and aggravate placental ischemia, which will promote the occurrence of PE [28]. Therefore, MMP2 and MMP9 have also been considered as biomarkers for predicting PE[29]. Studies have shown that activation of MMP9 transcription can promote the invasion and migration of HTR-8/SVneo cells [30]. Down-regulation of MMPs expression and (epithelial-mesenchymal transition) EMT process inhibits trophoblast invasion and migration [31]. In the current study, we found that overexpression of BMP9 up-regulated the protein levels of MMP-2, MMP-9 and N-cadherin, as well as decreased the protein level of E-cadherin. Inhibition of BMP9 has the opposite effect. This result suggests that BMP9 may have the properties of promoting the invasion and migration of trophoblast cells and regulate the occurrence of PE.

SDF1-mediated activation of CXCR4 is involved in hematopoiesis, immune response and organ development, but also in vascular remodeling or neovascularization, thereby regulating the occurrence of various diseases [32]. It has been reported that SDF-1α/CXCR4 and VEGF expression is significantly reduced in the placental bed of PE patients in the third trimester, which may be related to the pathogenesis of PE [33]. Studies have shown that overexpression of SDF1 and CXCR4 promotes cell migration and invasion of HTR-8/SVneo [34]. Coincidentally, the trophoblast-derived chemokine SDF1 promoted the invasion of human decidual stromal cells in early pregnancy by interacting with CXCR4 [35]. SDF1/CXCR4 signaling enhances crosstalk at the maternal-fetal interface by regulating invasion and placental angiogenic processes, affecting multiple pregnancy-related diseases including PE [36]. Furthermore, the SDF1/CXCR4 axis is involved in the regulation of vascular homeostasis. In the mode of interaction between endothelial progenitor cells and smooth muscle cells, SDF1/CXCR4 is involved in the proliferation and migration of mature endothelial cells, and affects focal smooth muscle cell accumulation and phenotypic transition [37]. Activation of SDF1/CXCR4 signaling promotes angiogenesis [38]. It is well known that insufficient trophoblast invasion and impaired endothelial function are hallmarks of PE. Therefore, targeting the SDF1/CXCR4 pathway to modulate trophoblast phenotype and endothelial cell function will help alleviate the progression of PE. In this study, we confirmed that the expression of SDF1 and CXCR4 was decreased in PE patient serum. Overexpression of BMP9 up-regulated the levels of SDF1 and CXCR4 in HTR-8/SVneo cells. Further mechanisms revealed that BMP9 promoted the migration and invasion of HTR-8/SVneo cells and inhibited apoptosis by activating the SDF1/CXCR4 pathway.

In conclusion, this study shows that BMP9 is downregulated in PE patient serum. Overexpression of BMP9 promoted the proliferation and migration of trophoblast cells, and knockdown of BMP9 inhibited the proliferation and migration of trophoblast cells. It suggests that BMP9 may be a biomarker molecule for PE and contribute to the diagnosis and treatment of PE. Mechanistic results showed that BMP9 up-regulated the levels of SDF1 and CXCR4. Overexpression of BMP9 promoted the migration and invasion of HTR-8/SVneo cells and inhibited apoptosis by activating the SDF1/CXCR4 pathway. However, the study of BMP9 in PE is still insufficient. In the future, we will focus on in vivo animal experiments to continue to verify the role of BMP9.

### Electronic supplementary material

Below is the link to the electronic supplementary material.

Supplementary Material 1: Research Hightlights.

Supplementary Material 2: Figure S1. Inhibiting CXCR4 reverses the effect of BMP9 on the proliferation, migration, and invasion of HTR-8/SVneo cells.

Supplementary Material 3: Figure S1.

## Data Availability

The datasets supporting the conclusions of this article are included within the article.
