# Supplementary material for: BMP9 maintains the phenotype of HTR-8/Svneo trophoblast cells by activating the SDF1/CXCR4 pathway
Source: BMC Mol Cell Biol. 2023 Aug 7;24:24. doi: 10.1186/s12860-023-00487-0 (PMC10405378; doi:10.1186/s12860-023-00487-0)
Supplement: Supplementary file 1 — Supplementary Material 1: Research Hightlights. [file 12860_2023_487_MOESM1_ESM.docx]

**Research Hightlights**

1. BMP9 promoted HTR-8/SVneo cell proliferation, migration and invasion
2. BMP9 inhibited apoptosis of HTR-8/SVneo cells
3. BMP9 positively regulated the expression of SDF1 and CXCR4
4. BMP9 regulated cellular phenotype and apoptosis through the SDF1/CXCR4 pathway.
