# Supplementary material for: BMP9 maintains the phenotype of HTR-8/Svneo trophoblast cells by activating the SDF1/CXCR4 pathway
Source: BMC Mol Cell Biol. 2023 Aug 7;24:24. doi: 10.1186/s12860-023-00487-0 (PMC10405378; doi:10.1186/s12860-023-00487-0)
Supplement: Supplementary file 2 — Supplementary Material 2: Figure S1. Inhibiting CXCR4 reverses the effect of BMP9 on the proliferation, migration, and invasion of HTR-8/SVneo cells. [file 12860_2023_487_MOESM2_ESM.docx]

**Figure S1. Inhibiting CXCR4 reverses the effect of BMP9 on the proliferation, migration, and invasion of HTR-8/SVneo cells.** After transfection of pcDNA3.1-BMP9 and si-CXCR4 plasmids for 24 h, the following indexes were measured. (A) RT-PCR was used to detect the expression of CXCR4. (B) The proliferation of HTR- 8/SVneo cells was evaluated by CCK8 assay. (C) Western blot was used to detect the expression levels of migration and invasion-related proteins (MMP-2, MMP-9, E-cadherin and N-cadherin). (D) The levels of apoptosis-related proteins (caspase-3, caspase-9, Bax and Bcl-2) were detected by western blot. ^*^ means *P<0.05* compared to control, ^#^ means *P<0.05* compared to ov-BMP9.
