# Supplementary figures and images for: BMP9 maintains the phenotype of HTR-8/Svneo trophoblast cells by activating the SDF1/CXCR4 pathway
Source: BMC Mol Cell Biol. 2023 Aug 7;24:24. doi: 10.1186/s12860-023-00487-0 (PMC10405378; doi:10.1186/s12860-023-00487-0)

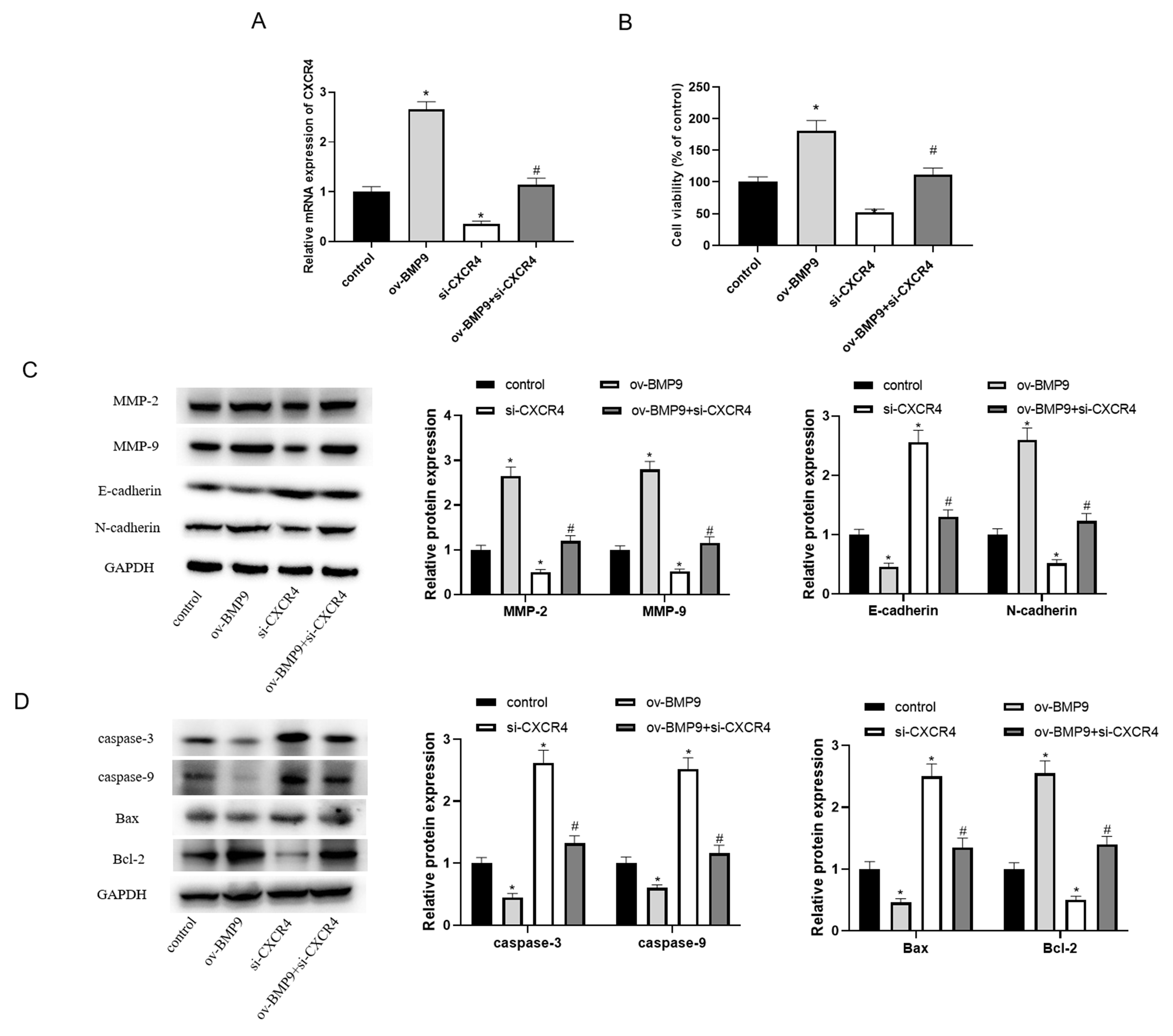

Supplement: Supplementary file 3 — Supplementary Material 3: Figure S1. [file 12860_2023_487_MOESM3_ESM.tif]
